# Effects of head-elevated position on tracheal intubation using a McGrath MAC videolaryngoscope in patients with a simulated difficult airway: a prospective randomized crossover study

**DOI:** 10.1186/s12871-022-01706-5

**Published:** 2022-05-30

**Authors:** Eun Hee Chun, Mi Hwa Chung, Jung Eun Kim, Kyung Mi Kim, Hye Sun Lee, Jung Mo Son, Jiho Park, Joo Hyun Jun

**Affiliations:** 1 Department of Anesthesiology and Pain Medicine, Kangnam Sacred Heart Hospital, Hallym University College of Medicine, 1, Shingil-ro, Yeongdeungpo-gu, Seoul, 07441 Republic of Korea; 2 Department of Anesthesiology and Pain Medicine, Asan Medical Center, University of Ulsan College of Medicine, Seoul, South Korea; 3 Department of Biostatistics, Yonsei University College of Medicine, Seoul, South Korea

**Keywords:** Laryngoscopy, videolaryngoscope, Macintosh blade, tracheal intubation

## Abstract

**Background:**

The head-elevated laryngoscopy position has been described to be optimal for intubation, particularly in obese patients and those with anticipated difficult airways. Horizontal alignment of the external auditory meatus and sternal notch (AM-S) can be used as endpoints for optimal positioning. Thus, we aligned the head-elevated position with the AM-S in the horizontal plane and evaluated its effect on laryngeal visualization and ease of intubation using a McGrath MAC videolaryngoscope in patients with a simulated difficult airway.

**Methods:**

Sixty-four patients were included in this prospective, crossover, randomized controlled trial. A cervical collar was used to restrict neck movement and mouth opening. The head-elevated position was achieved by raising the back section of the operation room table and ensuring that the end point was horizontally aligned with the AM-S (table-ramp method). The laryngeal view was randomly assessed in both head-flat and head-elevated positions based on the percentage of glottic opening (POGO) score and modified Cormack–Lehane (MCL) grade. External laryngeal manipulation was not permitted when laryngeal visualization was scored. The trachea was intubated only once (in the second position). The ease of intubation was assessed based on the need for optimization maneuvers, intubation difficulty scale (IDS) scores and time to intubation.

**Results:**

The mean table-ramp angle required to achieve the horizontal alignment of AM-S was 17.5 ± 4.1°. The mean POGO score improved significantly in the head-elevated position (59.4 ± 23.8%) when compared with the head-flat position (37.5 ± 24%) (*P* <  0.0001). MCL grade 1 or 2a was achieved in 56 (85.9%) and 28 (43.7%) of patients in the head-elevated and head-flat positions, respectively (P <  0.0001). Optimization maneuvers for intubation were required in 7 (21.9%) and 17 (53.1%) patients in the head-elevated and head-flat positions, respectively (*P* <  0.0001). The IDS scores and time to intubation did not differ significantly between the two positions.

**Conclusion:**

In the head-elevated position, aligning the AM-S in the horizontal plane consistently improved laryngeal visualization without worsening the view when the McGrath MAC videolaryngoscope was used in patients with simulated difficult airways. It also improved the ease of intubation, which reduced the need for optimization maneuvers.

**Trial registration:**

This trial was registered with www.clinicaltrials.gov, NCT04716218, on 20/01/2021.

**Supplementary Information:**

The online version contains supplementary material available at 10.1186/s12871-022-01706-5.

## Background

Videolaryngoscopes (VLs) can enhance a laryngeal view by providing indirect visualization of glottic opening and is recommend during tracheal intubation of patients with conditions that can make direct laryngoscopy (DL) difficult or impossible [1–3]. The McGrath MAC VL (Aircraft Medical Ltd., Edinburgh, UK) is a recently developed VL with a Macintosh-geometry blade (Mac-VL). These devices have the same blade as a standard laryngoscope. Thus, Mac-VL can be used to perform both indirect and direct laryngoscopy. The tracheal tube (TT) is inserted in the same manner as in DL, with or without a stylet [4–6]. However, as Mac-VLs lack the capacity to ‘see around corner’ achieved by hyper-angulated blade, they might provide only marginal marginally improve the view without clinically dramatic improvement when DL fails to provide sufficient laryngeal visualization [7, 8].

Optimal positioning of the head and neck is essential to achieve adequate laryngeal visualization during laryngoscopy [9]. The head elevated laryngoscopy position (HELP), which elevates the patient’s head and neck beyond the sniffing position, has been described to improve laryngeal view in obese patients and those with anticipated difficult airways [10, 11]. This position is typically attained by placing blankets beneath the patient’s head and shoulders, but it can be achieved by raising the back portion of the flat operating room (OR) table (table-ramp method) [10]. Studies are consistent in reporting that HELP improves preoxygenation, which prolongs apnea time when compared with the supine position [12–14]. However, there have been discordant results regarding head elevation height and better laryngeal exposure according to racial variations [11, 15]. Meanwhile, Greenland et al. [16] suggests that, through magnetic resonance imaging, raising the head until the external auditory meatus and sternal notch (AM-S) are in the horizonal plane leads to better anatomic alignment of the upper airway during laryngoscopy [16–18]. These findings support the use of individualized head elevation for optimal laryngeal exposure, which depends on the anatomy of the head and neck and the configuration of the chest [19, 20].

Thus, we hypothesized that the laryngeal exposure using McGrath MAC VL would be consistently improved in most patients with difficult airways when the HELP is applied to align the AM-S in the horizontal plane. The primary aim of this study was to determine whether the HELP using individual table-ramp angle as determined by the horizontal alignment of AM-S improve laryngeal visualization in simulated difficult airway with a cervical collar to restrict neck movement and mouth opening. The secondary aim was to evaluate the effect of this individualized approach on the ease of tracheal intubation using McGrath MAC VL.

## Methods

This was a prospective, crossover, randomized controlled trial of a simulated difficult airway with concurrent limited mouth opening and neck movement. Ethical approval for this study was approved by the institutional review board of Hallym University Kangnam Sacred Heart Hospital (approval number: 2020–09-009). The trial was registered prior to patient enrollment at www.clinicaltrials.gov (NCT04716218) on 20/01/2021 and conducted and reported according to the Consolidating Standards of Reporting Trials (CONSORT) 2010 statement [21]. This study was conducted from January 2021 to March 2021. We recruited 64 adult patients who received tracheal intubation under general anesthesia before elective surgery. All patients provided written informed consent and had an ASA physical status of I–III. The exclusion criteria were aspiration risk, bleeding tendency, uncontrolled hypertension, obstructive sleep apnea, and a known or anticipated difficult airway (e.g., Mallampati class III–IV, body mass index > 35 kg/m^2^, interincisor distance < 3.5 cm, thyromental distance < 6 cm).

### Study protocol

A McGrath™ MAC VL (Aircraft Medical, Ltd., Edinburgh, UK) with a curved blade and a camera was set up according to the standard practice at our institution. Tracheal intubation was performed using a size 3 McGrath™ MAC VL blade and a TT with an internal diameter of 6.5 mm for females and 7.5 mm for males (Mallinckrodt, St. Louis, MO, USA). A malleable stylet was not initially used to facilitate tracheal intubation because the McGrath MAC VL had a Macintosh-geometry blade [5, 22].

Using a computer-generated randomization table (www.randomizer.org), the enrolled patients were randomly assigned to one of the two sequences (Sequence 1, the head-elevated position was used first, followed by the head-flat; Sequence 2, the head-flat position was used first, followed by the head-elevated position). The sequence allocation was concealed in an opaque envelope, which was opened by the investigator responsible for randomization immediately before the initiation of anesthesia.

**Fig. 1:**
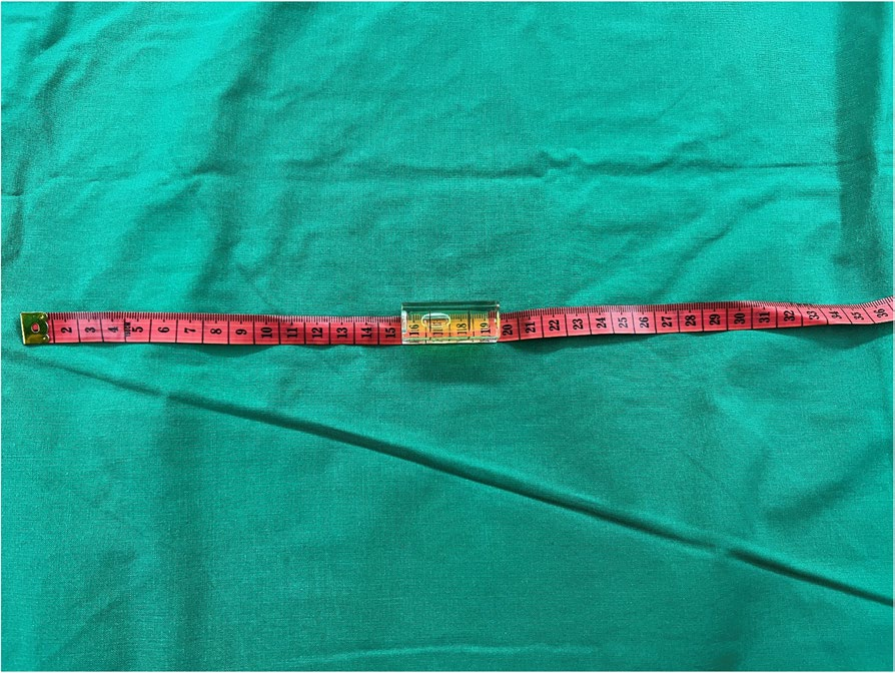
Spirit Level-Scale Assembly. A spirit level is attached to a measuring scale

**Fig. 2:**
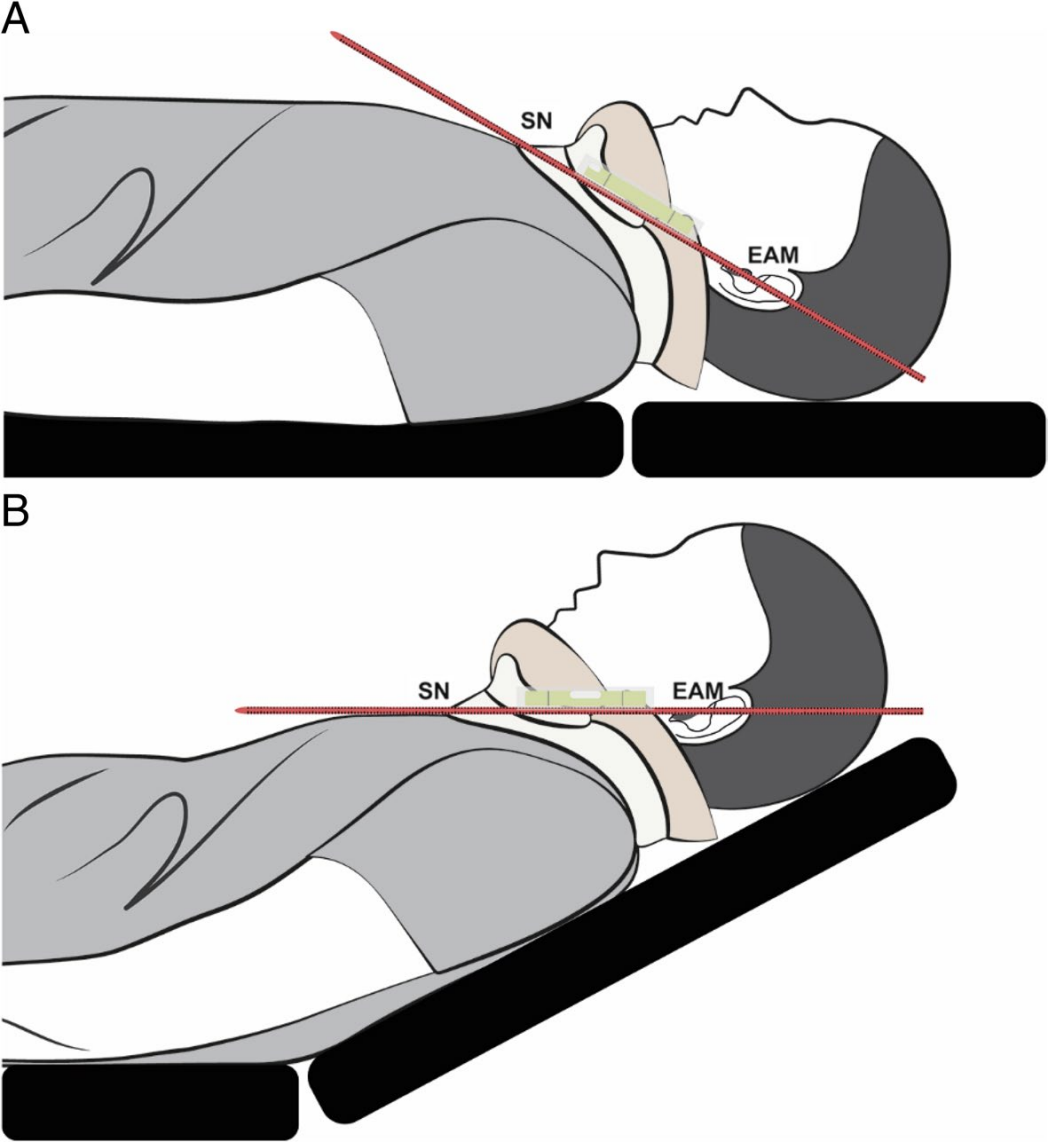
**A** Head-flat position without pillow. **B** Head-elevated position with horizontal alignment of the external auditory meatus (EAM) and sternal notch (SN). The position of the air bubble in the spirit level acts as a guide to adjust the table ramp angle

All patients were premedicated with glycopyrrolate (0.2 mg) at least 30 min before the initiation of anesthesia. After entering the operating room, each patient was positioned head-flat on the OR table, without a pillow under the head. An observer who was unaware of the sequence assignment assessed the airway based on the Mallampati class, thyromental distance, and interincisor distance at maximal mouth opening. Then, a semirigid cervical collar (Philadelphia Cervical Collar Co., Thorofare, NJ, USA) of appropriate size was placed around the patient’s neck, and the interincisor distance at maximal mouth opening was remeasured. The head-elevated position was achieved by raising the back section of the flat OR table with the end point being the horizontal alignment of the AM-S, which was objectively determined by the Spit Level-Scale assembly (Fig. 1). The table-ramp angle was adjusted to bring the air bubble in the spirit level to the center and measured using a digital protractor (Fig. 2) [23].

Anesthesia was induced with propofol (1.5 mg/kg) and remifentanil (0.5–1 μg/kg), followed by rocuronium (0.6 mg/kg). After verifying neuromuscular blockade using a nerve stimulator, tracheal intubation was performed using the McGrath MAC VL according to the assigned sequence. After the laryngeal view was assessed in the first position (end of period 1), the patient was ventilated using a facemask for 1 minute. The patient was then placed in the second position (start of period 2). Thus, the laryngeal view was assessed in the head-flat and head-elevated positions, but the trachea was intubated only once (in the second position). External laryngeal manipulations (ELMs) were not permitted when laryngeal visualization was assessed. To eliminate interobserver variation, a single experienced anesthesiologist (> 100 previous tracheal intubations using the McGrath MAC VL) performed all VL procedures.

### Outcome measurement

The following data were collected by two investigators (Eun Hee Chun and Joo Hyun Jun) who were uninvolved in the VL procedures.

The primary outcome was the laryngeal view in the head-flat and head-elevated positions (periods 1 and 2, respectively), which was assessed according to the percentage of glottic opening (POGO) score and modified Cormack–Lehane (MCL) grade. The POGO score reflects the proportion of the glottic area that is visible: a score of 100% denotes visualization of the whole glottis, from the interarytenoid notch to the anterior commissure, whereas a score of 0% denotes visualization of none of the glottis [24]. Based on the MCL grade, the laryngeal view was classified as easy (laryngeal inlet visible; MCL grade 1 or 2a), restricted (posterior glottic structures or epiglottis visible, where the latter could be lifted; MCL grade 2b or 3a), and difficult (epiglottis could not be lifted or no laryngeal structures visible; MCL grade 3b or 4) [25]. The data collection form included illustrations of the MCL grades and POGO scores to promote standardization.

The secondary outcome was the ease of intubation, which was measured in the second position (period 2). To evaluate this outcome, we recorded the optimization maneuvers used for successful tracheal intubation, such as withdrawing and reinserting the blade, increasing the lifting force, applying ELMs, bending the TT into a steeper curve, adding a stylet, or rotating the TT during passage into the trachea to avoid impacting the anterior wall of the subglottic space [26, 27]. The time to intubation, intubation difficult scale (IDS) score [28], reasons for failed intubation, and adverse effects were also recorded. Time to intubation represented the time between insertion into and removal of the VL blade from the mouth.

### Statistical analysis

Statistical analyses were performed using SAS (version 9.4; SAS Institute, Cary, NC, USA), SPSS (version 27.0; IBM Corp., Armonk, NY, USA), and R software (version 4.1.0; http://www.R-project.org). The distribution of continuous data was evaluated using the Shapiro–Wilk test. Normally distributed continuous variables are provided as the mean ± standard deviation and were analyzed using paired and independent *t tests*. Nonnormally distributed continuous variables are provided as the median (interquartile range) and were analyzed using Wilcoxon’s signed rank and Mann–Whitney U tests. Categorical data are expressed as *n* (%) for proportions and were compared using the McNemar and χ^2^ tests, with bootstrapping applied as appropriate. To evaluate possible carryover effects, the sum of the POGO scores in the first position (period 1) and second position (period 2) was calculated for each subject and compared across the two sequences using the unpaired *t* test. To evaluate possible period effects, the difference in POGO scores between the two periods was calculated for each subject and compared across the two sequences using the unpaired *t test* [29]. In all analyses, *P* <  0.05 was considered statistically significant.

We calculated the sample size based on the POGO score using PASS software (version 15.0; NCSS, LLC, Kaysville, UT, USA). Assuming that a 20% difference in POGO score was clinically important (standard deviation of 35%) [30], we determined that 28 pairs were required in each sequence for two-sided testing in this 2 × 2 crossover study, with 95% power and an alpha level of 5%. Therefore, we enrolled 32 participants in each sequence group, assuming a dropout rate of 10%.

## Results

**Fig. 3:**
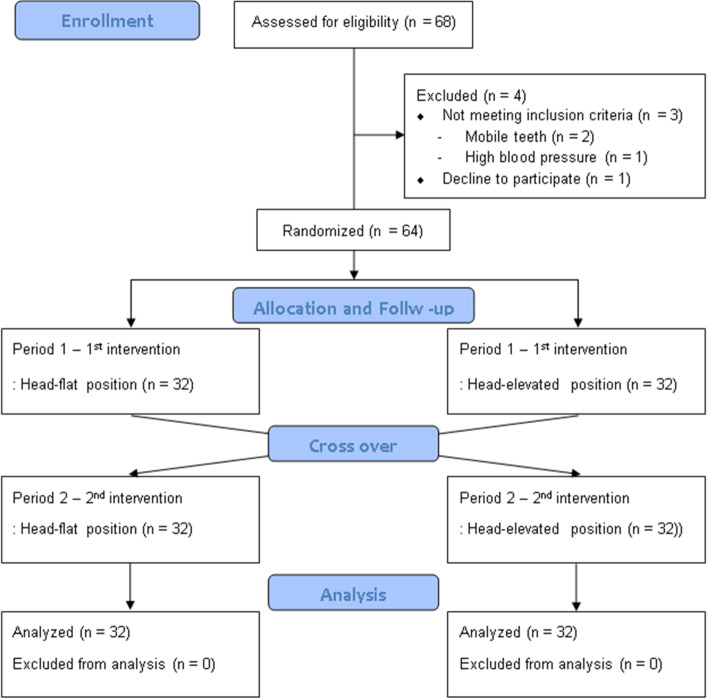
Study flow chart

In total, 68 potential participants were screened between January 2021 and March 2021. Three patients were excluded because of mobile teeth (*n* = 2) or uncontrolled hypertension (*n* = 1), and one declined to participate in the study. The remaining 64 patients were randomized (32 per group) and included in the analyses (Fig. 3). Table 1 shows the characteristics of the patients (including the patients’ airways). Neck motion was severely restricted after the cervical collar was applied, and the median interincisor distance at maximal mouth opening decreased from 40 (40 to 50) to 32 (30 to 35) mm, which was a significant difference (*P* < 0.0001). The mean table-ramp angle for horizontal alignment of the AM-S in the head-elevated position was 17.5 ± 4.1°.

**Table 1:** Baseline patient and airway characteristics

	Overall (*n* = 64)	Sequence 1^a^ (*n* = 32)	Sequence 2^b^ (*n* = 32)
Age, years	52.7 ± 13.9	53.4 ± 14.9	51.9 ± 13.0
Male/female	38/26	20/12	18/14
BMI, kg/m^2^	24.2 ± 2.9	23.6 ± 2.8	24.8 ± 2.8
ASA I/II/III	1/52/11	1/27/4	0/25/7
Mallampati class I/II	26/38	13/19	13/19
TMD, cm	8 (7 to 9)	8 (7 to 9)	8 (7 to 9)
ID without collar, mm	40 (40 to 50)	43 (38 to 50)	40 (40 to 48)
ID with collar, mm	32 (30 to 40)	30 (30 to 40)	35 (30 to 39)
Table-ramp angle, °	17.5 ± 4.1	17.1 ± 4.2	17.8 ± 4.1

Data are presented as the mean ± standard deviation, *n*, or median (interquartile range). ^a^Sequence 1: head-elevated position, followed by head-flat position. ^b^Sequence 2: head-flat position, followed by head-elevated position. *BMI* Body mass index, *TMD* Thyromental distance, *ID* Interincisor distance at maximal mouth opening

### Primary outcome

**Fig. 4:**
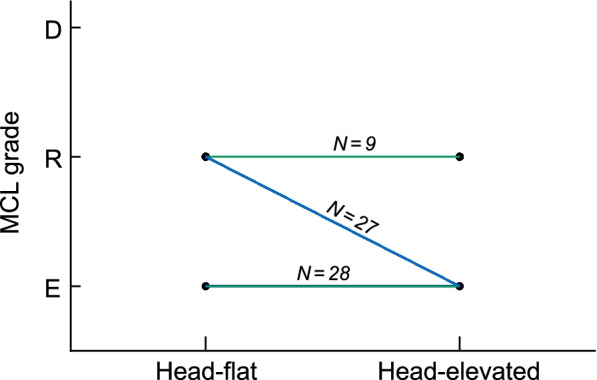
Laryngeal view based on modified Cormack–Lehane (MCL) grades in the head-flat and head-elevated positions. E = easy laryngeal view (MCL grade of 1 or 2a); R = restricted laryngeal view (MCL grade of 2b or 3a); D = difficult laryngeal view (MCL grade of 3b or 4); Blue lines = improved laryngeal view in the head-elevated position; green lines = no difference in laryngeal view between positions

**Fig. 5:**
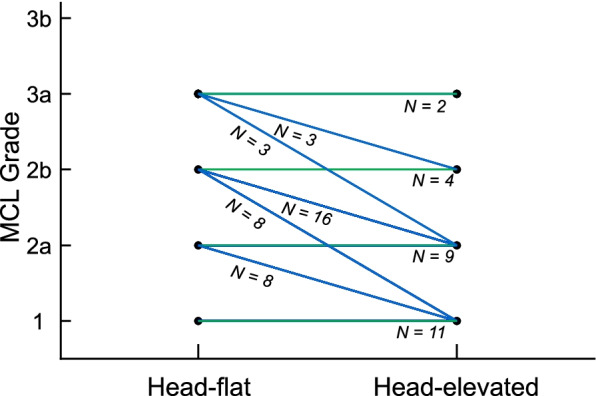
Changes in the modified Cormack–Lehane (MCL) grade in the head-elevated position compared to the head-flat position. Blue lines = improved MCL grade in the head-elevated position; green lines = no difference in MCL grade between positions. MCL grade did not worsen in the head-elevated position in any patient

Compared with the head-flat position, the head-elevated position provided significantly better or similar laryngeal visualization. The mean POGO score improved significantly in the head-elevated position (59.4 ± 23.8%) when compared with the head-flat position (37.5 ± 24%) (difference in means, 21.9%; 95% confidence interval [CI] 17.1 to 26.7%; P < 0.0001). An easy laryngeal view (MCL grade 1 or 2a) was achieved in 56 patients (85.9%) in the head-elevated position and only 28 (43.7%) in the head-flat position (difference in proportions, 42.2%; 95% CI 30 to 54.4%; P < 0.0001) (Fig. 4, Table 2). In 38 of 64 patients (59.4%), the head-elevated position improved the glottic view by one or two MCL grades, while the MCL grade was similar between positions in the remaining 26 patients (40.6%). The head-elevated position did not worsen the MCL grade in any patient (Fig. 5, Additional file 1).

**Table 2:** POGO scores and MCL grades in the head-flat and head-elevated positions

	Head-flat position (*n* = 64)	Head-elevated position (*n* = 64)	Difference (95% CI)	*P value*
POGO score, %	37.5 ± 24	59.4 ± 23.8	21.9 (17.1 to 26.7)	< 0.0001
MCL grade				
Easy: Grade 1/2a	11/17 (43.7)	27/28 (85.9)	42.2 (30 to 54.4)	< 0.0001
Restricted: Grade 2b/3a	28/8 (56.3)	7/2 (14.1)		–
Difficult: Grade 3b/4	0/0 (0)	0/0 (0)		–

Data are presented as the mean ± standard deviation or *n* (%). *CI* Confidence interval, *POGO* Percentage of glottic opening, *MCL* Grade, modified Cormack–Lehane grade

### Secondary outcome

The results of intubation in the second position (period 2) are summarized in Table 3. Intubation was successful on the first attempt in all patients. The proportion of patients requiring optimization maneuver(s) for tracheal intubation was higher in the head-flat position (*n* = 17; 53%) than in the head-elevated position (*n* = 7; 22%) (*P* = 0.01). There were no significant differences in median time to intubation (*P* = 0.11) or median IDS score (*P* = 0.13) between the two positions. Slight mucosal bleeding attributed to laryngoscopy or tracheal intubation was noted in one patient. No major complications (e.g., dental damage and palatal perforation) were observed.

**Table 3:** Ease of intubation in the second position (during period 2)

	Head-flat position (*n* = 32)	Head-elevated position (*n* = 32)	*P value*
Patients requiring optimization maneuvers	17 (53.1)	7 (21.9)	0.01
Blade withdrawal	2 (6.2)	2 (6.2)	
External laryngeal manipulation	11 (34.4)	7 (21.9)	
Increased lifting force	7 (21.9)	1 (3.1)	
Tracheal tube bending	2 (6.2)	1 (3.1)	
Stylet added	4 (12.5)	2 (6.2)	
Tracheal tube rotation	1 (3.1)	0 (0)	
Time to intubation, s	26.6 (22.1 to 32.7)	22 (19.4 to 30.1)	0.11
Intubation Difficulty Scale score	1 (1 to 3)	1 (0.5 to 1)	0.13

Data are presented as *n* (%) or median (interquartile range)

### Carryover and period effect

There was no apparent carryover effect for the primary outcome (POGO score; *P* = 0.32). However, a period effect was observed for the POGO score (*P* = 0.03) (Table 4). Although the difference in POGO scores between the head-flat and head-elevated positions was lower in period 2 than in period 1, the POGO scores remained better in the head-elevated position than in the head-flat position during period 2 (*P* = 0.008) (additional file 2).

**Table 4:** Carryover and period effects

	Sequence 1^a^ (*n* = 32)	Sequence 2^b^ (*n* = 32)	Difference (95% CI)	*P value*
POGO score, %				
Sequence effect^c^	102.3 ± 52.1	91.4 ± 33.4	10.9 (−11.0 to 32.9)	0.32
Period effect^d^	16.4 ± 16.3	27.3 ± 20.4	−10.9 (−20.2 to − 1.7)	0.02

Data are presented as the mean ± standard deviation. ^a^Sequence 1: head-elevated position, followed by head-flat position. ^b^Sequence 2: head-flat position, followed by head-elevated position. ^c^Sequence effect: sum of POGO scores in the first position (period 1) and second position (period 2). ^d^Period effect: difference between POGO scores in the head-elevated and head-flat positions (head-elevated minus head-flat). *CI* Confidence interval, *POGO* Percentage of glottic opening

## Discussion

This study demonstrated that the head-elevated position using an individual table-ramp angle (17.5 ± 4.1°) to align the AM-S in the horizontal plane consistently improved visualization of the larynx, as assessed by the POGO score and MCL grade, when using the McGrath MAC VL in patients with simulated concurrent limited neck movement and reduced mouth opening. Specifically, the mean POGO score improved from 37.5 ± 24% to 59.4 ± 23.8%, and the proportion of patients with an easy (MCL grade 1 or 2a) view increased from 43.7 to 85.9%. This individualized approach was also associated with reduced use of optimization procedures during McGrath MAC VL guided intubation. In the current study, these maneuvers were used in only 21.9% of patients in the head-elevated position compared to 53.1% of patients in the head-flat position. However, the time to intubation and IDS score did not differ significantly between the two positions.

Some authors have suggested that a higher head-elevated position is associated with further improvement in the laryngeal view in most patients [11, 31]. However, the mean table-ramp angle for aligning the AM-S in our study was 17.5 ± 4.1°, which was lower than the commonly suggested 25 degrees for improved laryngeal exposure [18, 32]. In a randomized study with a crossover design of 40 nonobese patients, Lee et al. found that the POGO was improved by approximately 25% in the head-elevated position when compared with the head-flat position [32]. However, in recent studies of large populations in the OR and intensive care unit, the results observed by Lee et al. could not be confirmed. That is, it was reported that the 25-degree head-elevated position did not improve or even worsen the glottic exposure [33, 34]. This discordant result can be explained by the fact that the head elevation height for optimal laryngeal exposure varies and depends on the anatomy of the head and neck and the configuration of the chest [19, 20]. Likewise, in another recent study [35], 3-, 6-, and 9-cm heights for the head-elevated position were compared with a crossover design of 50 patients. The best laryngeal view was achieved with 9-cm head elevation in most patients. However, in 5 patients who had short necks, the laryngeal view was better with a lower head-elevation height. Unlike previous studies that applied a fixed head elevation height, our study applied an individualized head elevation determined by the individual’s anatomical landmark, which may have contributed to consistent improvements of the laryngeal view without worsening in any of our patients.

There are several types of VL with different device designs and blade geometries. Each VL has unique features that may be advantageous under some conditions but disadvantageous under others [5]. In the same difficult airway scenario as in our study (application of a cervical collar to limit mouth opening and neck movement), Bathory et al. [36] reported an excellent laryngeal view (MCL grade 2a or higher in most patients) using a VL with a hyperangulated blade (HA-VL). This difference can be explained by the fact that the hyperangulated blade improves the capacity to see ‘around the corner’, allowing a view of glottic structures that are beyond the reach of Macintosh-style blades, with only minimal head and neck manipulation [5]. However, in the described setting, the initial placement of the HA-VL and subsequent guidance of the TT through the glottis might be hindered because of the small mouth opening [37, 38]. Furthermore, a HA-VL requires that a styletted TT be bent at an acute angle to match the blade’s curvature, which can contribute to a prolonged time to intubation and an increased risk of soft-tissue injury, such as oropharyngeal perforation [8, 39, 40]. In contrast, the lower angulation of the Mac-VL provides more room for TT manipulation, which leads to easier intubation and a reduced risk of trauma [41]. In our study, the head-elevated position to align the AM-S in the horizontal plane helped achieve an optimal laryngeal view with minimal head and neck manipulation (MCL grade 2a or higher in 85.9% of patients), even using McGrath MAC VL with a Macintosh-type blade.

Importantly, when using VLs, an improved laryngeal view does not automatically suggest that the tracheal intubation will be successful [42, 43]. For optimal laryngeal visualization with DL, reducing the angle between the oral axis and pharyngeal axis is necessary to permit a direct line of sight, which creates a relatively straight path for the TT. However, the indirect view of the glottis achieved with a camera on a curved blade of VL eliminates the need to necessarily align these axes. Thus, the tip of the TT during indirect laryngoscopy with VL must pass around an acute angle to enter the larynx, which complicates TT insertion and makes intubation difficult [5]. To compensate for their geometric limitations, some authors have suggested deliberate worsening of the view (by withdrawing the VL blade) to reduce the TT introduction angle, which thereby facilitates intubation [22]. However, this strategy negates the perceived advantage of VL, i.e., a good laryngeal view. In contrast, in our study, placing the patient in the head-elevated position created relatively straight passage of the TT and made tracheal intubation easier, according to the need for optimization maneuvers without compromising laryngeal visualization.

This study has some limitations. First, we enrolled patients with a simulated difficult airway. Although the use of a cervical collar is common when simulating difficult airways for research purposes [38, 44, 45], caution is required when extrapolating the results to genuine difficult airways. However, we did not enroll patients with genuine difficult airways because this is a rare and possibly life-threatening condition [46]. Second, as our study was specifically performed for limited neck extension and mouth opening, our results may not be applicable to other types of difficult airways. Third, all laryngoscopy/intubation procedures were performed by a single anesthesiologist who was experienced in simulated difficult airways. Caution is required when extrapolating our results to less experienced physicians and/or patients with naturally difficult airways. Finally, as with any crossover study, a carryover effect from the first to the second period was possible. Stress-induced relaxation of the tongue and pharyngeal tissues occurs during laryngoscopy, so we randomized the position order to reduce the influence of this phenomenon. Furthermore, our analysis implied that there were no significant carryover effects.

## Conclusions

Compared with a head-flat position, the use of the head-elevated position to align the AM-S in the horizontal plane consistently improved laryngeal visualization without worsening the view when using a McGrath MAC VL in patients with simulated concurrent limited neck extension and mouth opening. The head-elevated position also improved the ease of intubation, as indicated by the proportion of patients for whom optimization maneuvers were used. Our results imply that the table-ramp angle to achieve this position needs to be individualized.

## Supplementary Information

**Additional file 1: Supplemental Table 1.** Differences in MCL grades between the head-elevated and head-flat positions.

**Additional file 2: Supplemental Table 2.** Possible effect of period 1 (first position) on period 2 (second position).

## Data Availability

The datasets generated and/or analyzed during the current study are not publicly available due to the regulation of the Institutional Review Board but are available from the corresponding author after obtaining permission from the IRB for sharing the dataset on reasonable request.

## Abbreviations

AM-S: Alignment of external auditory meatus and sternal notch
CI: Confidence interval
DL: Direct laryngoscopy
EAM: External auditory meatus
ELMs: External laryngeal manipulations
HA-VL: Hyperangulated videolaryngoscope
IDS: Intubation Difficulty Scale
Mac-VL: Macintosh-geometry videolaryngoscope
MCL: Modified Cormack–Lehane
SN: Sternal notch
POGO: Percentage of glottic opening
TT: Tracheal tube
VL: Videolaryngoscope
