# Supplementary material for: Effects of head-elevated position on tracheal intubation using a McGrath MAC videolaryngoscope in patients with a simulated difficult airway: a prospective randomized crossover study
Source: BMC Anesthesiol. 2022 May 30;22:166. doi: 10.1186/s12871-022-01706-5 (PMC9150377; doi:10.1186/s12871-022-01706-5)
Supplement: Supplementary file 1 — Additional file 1: Supplemental Table 1. Differences in MCL grades between the head-elevated and head-flat positions. [file 12871_2022_1706_MOESM1_ESM.docx]

**Supplemental Table 1.** Differences in MCL grades between the head-elevated and head-flat positions

|  | Head-flat position | | | |  |
| --- | --- | --- | --- | --- | --- |
|  | 1 | 2a | 2b | 3 |  |
| Head-elevated position |  |  |  |  |  |
| 1 | 11‡ | 8* | 8† | 0 | 27 (42%) |
| 2a | 0 | 9‡ | 16* | 3† | 28 (44%) |
| 2b | 0 | 0 | 4‡ | 3* | 7 (11%) |
| 3 | 0 | 0 | 0 | 2‡ | 2 (3%) |
|  | 11 (17%) | 17 (27%) | 28 (44%) | 8 (13%) |  |

Data are presented as *n* or *n* (%). MCL grade, modified Cormack–Lehane grade.

*The head-elevated position improved the glottic view by one MCL grade.

†The head-elevated position improved the glottic view by two MCL grades.

‡No difference in MCL grade between positions.
