# Supplementary material for: Effects of head-elevated position on tracheal intubation using a McGrath MAC videolaryngoscope in patients with a simulated difficult airway: a prospective randomized crossover study
Source: BMC Anesthesiol. 2022 May 30;22:166. doi: 10.1186/s12871-022-01706-5 (PMC9150377; doi:10.1186/s12871-022-01706-5)
Supplement: Supplementary file 2 — Additional file 2: Supplemental Table 2. Possible effect of period 1 (first position) on period 2 (second position). [file 12871_2022_1706_MOESM2_ESM.docx]

| **Supplemental Table 2.** Possible effect of period 1 (first position) on period 2 (second position) | | | | |
| --- | --- | --- | --- | --- |
| Period 1 | Head-flat  (*n* = 32) | Head-elevated  (*n* = 32) | Difference  (95% CI) | *P*-value |
| POGO score (%) | 32.0 ±19.3 | 59.4 ± 27.5 | 27.3 (15.5 to 39.2) | < 0.0001 |
| Period 2 | Head-flat  (*n* = 32) | Head-elevated  (*n* = 32) | Difference  (95% CI) | *P*-value |
| POGO score (%), | 43.0 ± 27.1 | 59.4 ± 19.8 | 16.4 (4.5 to 28.3) | 0.008 |

Data are presented as mean ± SD. POGO, percentage of glottic opening
